# Changes in foot posture and function following total knee replacement surgery

**DOI:** 10.1186/1757-1146-5-S1-P17

**Published:** 2012-04-10

**Authors:** Pazit Levinger, Hylton B Menz, Adam D Morrow, Julian A Feller, John R Bartlett, Mohammad R Fotoohabadi, Neil Bergman

**Affiliations:** 1 Musculoskeletal Research Centre, La Trobe University, Melbourne, VIC, 3086 Australia; 2 Warringal Medical Centre, Melbourne, Vic, 3084, Australia

## Background

Knee malalignment and variations in foot posture and function affect the forces transmitted through the knee joint and are associated with knee pain and medial tibiofemoral cartilage damage [1]. However, it is unclear whether altered foot posture and function are a compensatory mechanism to accommodate knee malalignment. Therefore, this study investigated changes in foot posture and function after realignment of the knee following total knee replacement (TKR) in people with medial compartment knee OA.

## Materials and methods

Nineteen patients (6 females and 13 males; mean age 67.5 ± 5.9 years, height 169.1 ± 9.9 cm, mass 87.9 ± 11.8 kg and BMI 31.0 ± 5.7 kg/m2) diagnosed with predominantly medial compartment knee OA who were scheduled for TKR surgery participated in the study, and were tested prior to and 12 months after TKR. Foot Posture Index (FPI) and arch index were measured as well as motion of the tibia, rearfoot and forefoot using a 3D motion analysis system incorporating a multisegment foot model (Oxford Foot Model).

## Results

Significant increases in tibial external rotation (-18.7± 7.0° vs -22.5 ± 8.7°, p = 0.002) and tibial transverse plane range of motion (ROM) (-9.1 ± 4.6° vs -11.4 ± 6.1°, p = 0.0028) were observed following the surgery. An increase in rearfoot ROM in the frontal plane (8.6 ± 2.6° vs 10.4 ± 2.7°, p = 0.002) and a decrease in rearfoot transverse plane ROM (8.7 ± 5.3° vs 5.9 ± 4.1°, p = 0.038) were observed. No significant differences were found between pre and post-surgery in the FPI and or the arch index.

## Conclusions

Following TKR, there is an increase in the ROM of the rearfoot in the frontal plane, but no change in static foot posture suggesting that rearfoot motion compensates for changes in the alignment of the knee.
